# Application of machine learning techniques to electron microscopic/spectroscopic image data analysis

**DOI:** 10.1093/jmicro/dfz036

**Published:** 2019-11-12

**Authors:** Shunsuke Muto, Motoki Shiga

**Affiliations:** 1 Electron Nanoscopy Division, Advanced Measurement Technology Center, Institute of Materials and Systems for Sustainability, Nagoya University, Furo-cho, Chikusa-ku, Nagoya 464-8603, Japan; 2 Faculty of Engineering, Gifu University, 1-1 Yanagido, Gifu, Gifu 501-1193, Japan; 3 PRESTO, Japan Science and Technology Agency, 4-1-8, Honcho, Kawaguchi-shi, Saitama 332-0012, Japan; 4 Center for Advanced Intelligence Project, RIKEN, Nihonbashi 1-chome Mitsui Building, 15th floor, 1-4-1 Nihonbashi, Chuo-ku, Tokyo 103-0027, Japan

**Keywords:** tensor decomposition, non-negative matrix factorization, scanning transmission electron microscopy, electron energy-loss spectroscopy, hyperspectral image analysis

## Abstract

The combination of scanning transmission electron microscopy (STEM) with analytical instruments has become one of the most indispensable analytical tools in materials science. A set of microscopic image/spectral intensities collected from many sampling points in a region of interest, in which multiple physical/chemical components may be spatially and spectrally entangled, could be expected to be a rich source of information about a material. To unfold such an entangled image comprising information and spectral features into its individual pure components would necessitate the use of statistical treatment based on informatics and statistics. These computer-aided schemes or techniques are referred to as multivariate curve resolution, blind source separation or hyperspectral image analysis, depending on their application fields, and are classified as a subset of machine learning. In this review, we introduce non-negative matrix factorization, one of these unfolding techniques, to solve a wide variety of problems associated with the analysis of materials, particularly those related to STEM, electron energy-loss spectroscopy and energy-dispersive X-ray spectroscopy. This review, which commences with the description of the basic concept, the advantages and drawbacks of the technique, presents several additional strategies to overcome existing problems and their extensions to more general tensor decomposition schemes for further flexible applications are described.

## Introduction

‘Artificial intelligence (AI)’, ‘machine learning (ML)’, and ‘deep learning (DL)’ are the keywords of the present special issue of *Microscopy*. These terms can be considered to be nested within one another: DL is a subset of ML, but ML can be a subset of AI, the last of which has become an umbrella term for any computer program (or a system) that does something smart. Among these terms, machine learning is vitally necessary for AI to train the system to become smart by extracting and summarizing massive training data sets. Needless to say, in terms of current analytical instruments and apparatus, the aid of a computer is indispensable for their operation and subsequent data analysis, and it should thus be considered that any attempts to introduce technical improvements in analytical science involve strategies that can, to a greater or lesser extent, be recognized as resorting under AI. On the other hand, ML is defined as the field of study that enables computers to learn without being explicitly programmed and is attributed to Arthur Samuel, who coined the term ‘machine learning’ in 1959 [1]. DL emerged more recently and belongs to the broader family of machine learning methods based on artificial neural networks. In this sense, this article focuses on data processing to extract enhanced information from the originally observed experimental data sets, a discipline currently often referred to as ‘informatics’ among ML or DL in the broader sense of the words.

In this article, the key equation is a simple bilinear matrix equation:

(1) \begin{equation*} \mathbf{Y} = \textbf{AX} + \mathbf{E} \end{equation*}

where **Y** represents experimentally detected signals, such as a two-dimensional (2D) image or a set of spectra in the field of electron microscopy, the main topic of interest here. The signals contained in **Y** are usually a mixture of (unknown) source signals **X** and noise **E**. **A** is referred to as an (also unknown) mixing matrix that plays the role of an instrumental function, filter or interface that mixes the source signals. Equation (1) provides a common thread in various approaches for noise removal, model reduction, signal reconstruction, and the purpose of blind source separation (BSS) is to replace the original data by a lower dimensional approximate representation obtained via a matrix or multi-way array factorization or decomposition that plays a fundamental role in enhancing the data and extracting latent components. It is, however, not possible to cover all the techniques that are currently available; thus, this article mainly focuses on several subsets of machine learning that are applied to solve a wide variety of problems related to electron microscopy and spectroscopy, particularly in the field of materials science. The application of ML to medical/biological fields, some of the most important fields in which AI has found application, is also beyond the scope of this article and is considered in other articles in this special issue.

In this article, we attempt to concentrate on physical insights and an intuitive understanding underlying the ideas of the concepts and techniques presented here at the cost of mathematical details. We assume readers to have a good background in elementary linear algebra at the university level and to be familiar with the principles of matrix arithmetic, matrix rank and principal component analysis, based on singular value decomposition by eigenvalue analysis.

## Non-negative matrix factorization for hyperspectral image analysis

**Fig. 1:**
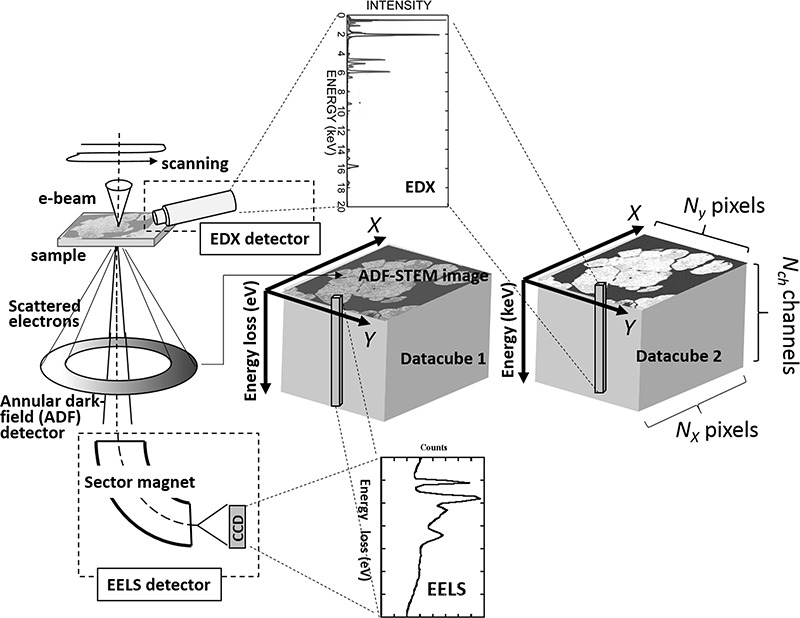
Schematic of STEM-EELS/EDXS HSIA. In this example, two HSI datacubes are concurrently obtained by a single scan of the ROI in the sample.

A ‘hyperspectral image (HSI)’ refers to a set of spectroscopic data sampled from a region of interest (ROI) of a specimen with a small step width by scanning using a probe tip, focused electrons, laser or infrared beam, which is a rich source of physical/chemical information of the specimen. The data set is sometimes known as a ‘datacube’, because of its three-dimensional structure, constructed by two-dimensional spatial coordination together with spectral channels as an energy or wavelength axis. Among others, we are interested in spectroscopic data, particularly obtained using scanning transmission electron microscopy (STEM) and the associated analytical methods of electron energy-loss spectroscopy (EELS) and energy-dispersive X-ray spectroscopy (EDXS), as schematically shown in Fig. 1. Each spectrum recorded from a specific position on the specimen reflects not only the fundamental physical/chemical states of the material but also the subtle changes associated with local defects at the particular location, which may overlap or overlay one another, depending on the size of the scanning probe and specimen structure. In this respect, it becomes possible to visualize each physical/chemical state involved as a 2D spatial distribution map in the specimen by isolating each latent component spectrum from the datacube obtained. This has been realized by a conventional multiple linear least square (MLLS) fit using a set of reference (standard) spectra as far as the component spectra are known and available in a database. The strategy of MLLS fitting, however, is no longer effective when the data set includes spectral components associated with unknown and/or hidden features that may be characterized by an extremely small signal-to-noise ratio. In spectroscopic data sets, the entries of which are all non-negative, the technique employed to isolate the constituent components is known as non-negative matrix factorization (NMF), a particular field among similar statistically unsupervised machine learning techniques such as hyperspectral image analysis (HSIA), multivariate curve resolution or BSS that can solve the aforementioned problem.

Different strategies are required for EDXS and EELS STEM-HSI because of the different features of their peaks: EDXS data consist of a number of discrete peaks with less background, whereas EELS data exhibit non-definite peak profiles with higher background intensity. In addition, the use of HSI to analyze EDXS data is intended to separate the different phases embedded in the data and to mine hidden small phases consisting of different sets of peaks (elements) with different relative intensities. On the other hand, EELS is typically used to separate different chemical states of the same element, where the peak profiles are extensively overlapped. In this respect, EELS analysis by NMF is more challenging and needs special care to lead to a physically plausible solution.

A comprehensive survey of models and algorithmic aspects for the general use of NMF and its various extensions and modifications has been published [2]. Readers who are interested in this field can find the mathematical details, many other associated techniques and algorithms, and the corresponding MATLAB source codes there. In addition, readers are referred to our GitHub repository where they would find several useful Python source codes we have developed and introduce below [3].

### Basic concepts of NMF

**Fig. 2:**
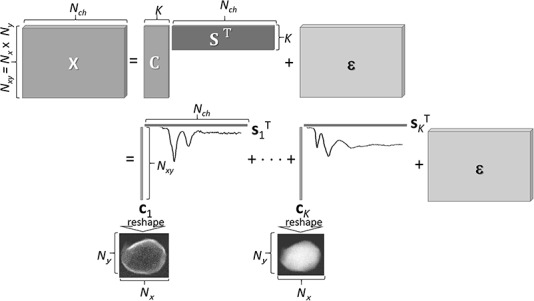
Illustration of matrix Eq. (2) (upper row) and its vector form Eq. (3) (bottom row). Entries in each **c***_j_* vector are reshaped back to the original 2D order, providing the spatial distribution of spectral component **s***_j_*.

Referring to Fig. 1, let $\mathbf{X}\in{\mathbb{R}}_{+}^{N_{xy}\times{N}_{ch}}$ be a data matrix, a simply expanded 2D matrix from the 3D datacube, where *N*_xy_ = *N_x_* × *N*_y_ and ${\mathbb{R}}_{+}$ is the set of all non-negative real numbers, referring to Fig. 1. NMF factorizes **X** into two thin matrices $\mathbf{C}=\Big[{\mathbf{c}}_1,\dots, {\mathbf{c}}_K\Big]\in{\mathbb{R}}_{+}^{N_{xy}\times K}$ and $\mathbf{S}=\Big[{\mathbf{s}}_1,\dots, {\mathbf{s}}_K\Big]\in{\mathbb{R}}_{+}^{N_{ch}\times K}$*,* where **c***_k_*$\in{\mathbb{R}}_{+}^{N_{xy}}$ and **s***_k_*$\in{\mathbb{R}}_{+}^{N_{ch}}$ are column vectors and the number of components *K* (*a priori* unknown; in other words, the number of pure chemical states, to be determined *a posteriori*. Further details appear in a subsequent section.) is much smaller than both *N*_xy_ and *N*_ch_. Thus, the factorization model has the same form as Eq. (1):

(2) \begin{equation*} \mathbf{X}=\mathbf{C}{\mathbf{S}}^{\mathrm{T}}+\boldsymbol{\varepsilon}, \end{equation*}

where the superscript T denotes the matrix (vector) transpose and $\boldsymbol{\varepsilon} \in{\mathbb{R}}^{N_{xy}\times{N}_{ch}}$ is a matrix of observation noise of which the elements are statistically independent of each other. In our problem setting, **X** is observed, whereas **C** and **S** are not observed. The goal of NMF is an ‘inverse problem’ to identify the optimal **C** and **S**, given experimental data **X** under a suitable noise model $\boldsymbol{\varepsilon}$, as schematically illustrated in the upper part of Fig. 2.

This NMF model can also be represented as the special form of a bilinear model as shown in the lower part of Fig. 2:

(3) \begin{equation*} \mathbf{X}=\sum_{k=1}^K{\mathbf{c}}_k{\mathbf{s}}_k^{\mathrm{T}}+\boldsymbol{\varepsilon} . \end{equation*}

Thus, we can build an approximate representation of the non-negative data matrix **X** as the sum of rank-one non-negative matrices ${\mathbf{c}}_k{\mathbf{s}}_k^{\mathrm{T}}$. Equation (3) clearly expresses the NMF model that, given an experimental data set, is used to search the optimal set of latent spectral basis (pure components or also endmembers) and its weight (relative fraction) at each sampling point. Once the solution of Eq. (2) is obtained, each column vector **c***_k_* is reshaped to the original 2D *N_x_* × *N*_y_ matrix form, representing the spatial abundance map of the spectral basis (signature of a certain chemical state) **s***_k_*, as shown in Fig. 2.

### ALS algorithm

As discussed in a subsequent section, the solution of Eq. (2) is not unique and the matter of central concern is how to attain the desired correct solution under the given constraint conditions. One of the most unambiguous methods to solve, Eq. (2) is principal component analysis (PCA), which is based on the singular value decomposition (SVD) algorithm [4]. PCA successively unfolds the components in the order of their variance, or the magnitude of the eigenvalues by SVD, in a way analogous to finding the principal axes of angular momentum of a rigid body in mechanics [5]. The obtained matrix **S** in Eq. (2) is, however, a purely mathematical product, with each of the column vectors, which are mutually orthogonal, often providing components with negative entries that make little physically interpretable sense. Nevertheless, PCA still remains useful for effectively screening minor components such as observation noise (unwanted random data) and providing a certain criterion for the number of significant components included in the data set [6–8]. In the field of signal processing, several techniques other than PCA, such as independent component analysis (ICA) [9,10], partial least squares [11] and canonical correlation analysis [12], are used, each imposing an additional constraint on the optimizing algorithm that depends on the nature of the solution specific to the problem of interest. In this respect, the key characteristic of HSIA that ensures that the solution is physically meaningful and interpretable is ‘non-negativity’.

The simplest way to solve the standard NMF model (2) is referred to as the alternating least squares (ALS) algorithm [2]. Unfortunately, the standard ALS algorithm and its simple modifications can be relatively slow when solving large-scale problems, often returning suboptimal solutions, which are quite sensitive with respect to noise.

Estimating the factor matrices **C** and **S** in Eq. (2) requires us to consider a measure to quantify the difference between the data matrix **X** and the approximation $\hat{\mathbf{X}}=\mathbf{C}{\mathbf{S}}^{\mathrm{T}}$. The similarity measure between **X** and $\hat{\mathbf{X}}$, also referred to as the distance, divergence or cost function minimization, depends on the probability distributions of noise. The simplest and most often used measure is based on the squared Euclidean distance or Frobenius norm, ${D}_F\Big(\Big.\mathbf{X}\Big\Vert \mathbf{C}{\mathbf{S}}^{\mathrm{T}}\Big)$:

(4) \begin{equation*} {D}_F\left(\left.\mathbf{X}\right\Vert \mathbf{C}{\mathbf{S}}^{\mathrm{T}}\right)=\frac{1}{2}{\left\Vert \mathbf{X}-\mathbf{C}{\mathbf{S}}^{\mathrm{T}}\right\Vert}_F^2. \end{equation*}

This measure assumes the Gaussian noise model. The ALS algorithm to minimize the cost function (4) can then be described by the following update rules, after initializing **C** randomly (or sometimes using a specific deterministic strategy):

(5) \begin{equation*} {\mathbf{S}}^{\mathrm{T}}\leftarrow{\left[{\left({\mathbf{C}}^{\mathrm{T}}\mathbf{C}\right)}^{-1}{\mathbf{C}}^{\mathrm{T}}\mathbf{X}\right]}_{+}, \end{equation*}

(6) \begin{equation*} \mathbf{C}\leftarrow{\left[\mathbf{XS}{\left({\mathbf{S}}^{\mathrm{T}}\mathbf{S}\right)}^{-1}\right]}_{+}, \end{equation*}

where [*x*]_+_ = max [*ε_0_*, *x*] is a half-wave rectifying nonlinear projection to enforce non-negativity or a positive constraint, and *ε_0_* is set to zero or a small constant. It should be noted that the cost function (4) is convex with respect to either the matrix **C** or **S**, but not both, and the ALS method is not guaranteed to converge to a global minimum and the solution is often not sufficiently accurate. One of the simplest modifications to escape from local minima by confirming stable updates during optimization of **C** or **S** is referred to as modified ALS (MALS), where a diagonal weight matrix $\boldsymbol{W}\in{\mathbb{R}}^{\boldsymbol{K}}\times{\mathbb{R}}^{\boldsymbol{K}}$ is introduced by adding *l*_2_ regularization terms used in a ridge-regression algorithm [13,14] to the original cost function of ALS. The update rules (5) and (6) of ALS are now modified as:

(7) \begin{equation*} {\mathbf{S}}^{\mathrm{T}}\leftarrow{\left[{\left({\mathbf{C}}^{\mathrm{T}}\mathbf{C}+{\mathbf{W}}_{\mathbf{S}}\right)}^{-1}\left({\mathbf{C}}^{\mathrm{T}}\mathbf{X}+{\mathbf{W}}_{\mathbf{S}}{\mathbf{S}}^{\mathrm{T}}\right)\right]}_{+}, \end{equation*}

(8) \begin{equation*} \mathbf{C}\leftarrow{\left[\left(\mathbf{XS}+{\mathbf{C}\mathbf{W}}_{\mathbf{C}}\right){\left({\mathbf{S}}^{\mathrm{T}}\mathbf{S}+{\mathbf{W}}_{\mathbf{C}}\right)}^{-1}\right]}_{+}. \end{equation*}

This modification allows the ALS updates to be stable against collinear data. A key feature in the implementation of MALS is weight adjustment. It is typically desirable to assign high weights in the very early iterations such that large adjustments are made. The weights are adjusted according to the difference in magnitude of negative values in successive iterative cycles between the current and previous estimates of the factors, a detailed discussion of which can be found in the literature [13].

The MALS algorithm is simple, relatively fast and the update rules can easily be modified at each step by imposing additional constraints, depending on the requirements of the problem of interest. We have applied the MALS method to solve a wide range of problems pertaining to materials and have obtained fruitful results particularly when analyzing the degradation of lithium-ion battery cathode materials that are subject to many charge-discharge cycles [15–19], probed by STEM-EELS.

### Non-uniqueness problem and initialization of NMF

The NMF method in general does not guarantee a unique solution (neglecting unavoidable scaling and permutation ambiguities). Consider the quadratic cost function (4) (neglecting the pre-factor):

(9) \begin{align*} {D}_F\left(\left.\mathbf{X}\right\Vert \mathbf{C}{\mathbf{S}}^{\mathrm{T}}\right)={\left\Vert \mathbf{X}-\mathbf{C}{\mathbf{S}}^{\mathrm{T}}\right\Vert}_F^2={\left\Vert \mathbf{X}-\mathbf{C}{\mathbf{R}}^{-1}\mathbf{R}{\mathbf{S}}^{\mathrm{T}}\right\Vert}_F^2\nonumber\\={\left\Vert \mathbf{X}-\hat{\mathbf{C}}{\hat{\mathbf{S}}}^{\mathrm{T}}\right\Vert}_F^2. \end{align*}

Although a rotational matrix **R** can be selected in many ways, as long as the rotated $\hat{\mathbf{C}}\ \Big(=\mathbf{C}{\mathbf{R}}^{-1}\Big)$ and ${\hat{\mathbf{S}}}^{\mathrm{T}}\ \Big(=\mathbf{R}{\mathbf{S}}^{\mathrm{T}}\Big)$ are non-negative, the costs are the same. It is sufficient to incorporate a certain degree of sparsity or smoothness constraints in the objective function for solving the NMF problem uniquely. This has been extensively discussed, and additional measures such as closure, unimodality, selectivity and local rank constraints have been imposed, depending on the prior information available in each specific problem [2,20].

When no prior information is available, it is helpful to mitigate the rotational ambiguity issues by normalizing the columns in **C** and/or the rows of **S**^T^. The columns **c***_j_* of **C** = [**c**_1_, …, **c***_k_*] are scaled in the following way:

(10) \begin{align*} \mathbf{C}\leftarrow \mathbf{C}{\mathbf{D}}_A,\mathrm{where}\ {\mathbf{D}}_A=\operatorname{diag}\left({\left\Vert{\mathbf{c}}_1\right\Vert}_p^{-1},{\left\Vert \nonumber{\mathbf{c}}_2\right\Vert}_p^{-1},\dots, {\left\Vert{\mathbf{c}}_k\right\Vert}_p^{-1}\right)\\ \,p\in \left[0,\infty \right). \end{align*}

Extensive empirical testing has led to the recognition that the best results can be obtained for *P* = 2, that is, when the columns of **C** are normalized to unit *l*_2_-norms. This may be justified by the fact that the mixing matrix should usually contain only a few dominant entries in each column, which is emphasized by the normalization to the unit *l*_2_-norm. This normalization for the ALS and MALS algorithms helps to mitigate numerical instabilities and ill-conditioning, although it complicates the process of searching for the global minimum. On the other hand, when normalization is applied to the rows of **S**^T^ that contain a single type of EEL spectrum [e.g. near-edge fine structure (ELNES)] from a single element, normalization to the unit *l*_2_-norm [the spectral intensity (the area subtended by the net spectrum above the background)] corresponds to normalization by the cross-section for the electron transition. This allows the interpretation of each entry in **C** to be the relative concentration of the corresponding spectral component at the spatial coordinate. Moreover, to avoid the rotational ambiguity of NMF, the rows of **S**^T^ should be sparse or zero grounded. The sparsity condition mostly holds for STEM-EDXSS or 3D-AP data sets, as opposed to STEM-EELS data sets, for which the sparsity condition barely holds. This problem is discussed in more detail in the next section. On the other hand, the zero-grounded condition can be easily achieved by removing the baseline or background from the input data **X**. Strictly speaking, the backgrounds in EELS and EDXS convey material information at the sampling points and are in principle inseparable from the core electron excitation/emission spectra. The removal of background signals can sometimes distort the information incorporated therein, and hence the unfolded spectral profiles are unnaturally biased. This effect often has serious consequences in practical applications and is discussed in a later section.

The solution provided by NMF algorithms usually highly depends on the initial conditions, i.e. its starting values. Poor initialization can result in slow convergence or entrapment in a local minimum far from the global minimum and can even lead to an incorrect or irrelevant solution. The following rule of thumb to obtain a stable optimization result was proposed [2]:

(i) First, generate *R* (typically10 or more) initial matrices **C** and **S**. This could be based either on random starts or on the output of the simplest ALS-NMF algorithm.
(ii) Run a specific NMF algorithm for each set of initial matrices and for *I*_init_ iterative cycles. As a result, the NMF algorithm provides *R* initial estimates of the matrices **C**^(*r*)^ and **S**^(*r*)^ accordingly.
(iii) Select the estimates corresponding to the lowest value of the cost function among the *R* trials as initial values for the final factorization.

The multi-start initialization thus selects the initial estimates for **C** and **S** to give the steepest decrease in the assumed objective function *D*(**X**||**CS**^T^) by alternating the steps for checking the convergence results after a number of initial alternating steps. The initial estimates **C** and **S**, which give the lowest values of *D*(**X**||**CS**^T^) after the alternating steps, are expected to be the most suitable candidates for the subsequent ALS optimization. The algorithm is generally considered to be quite efficient when the number of steps exceeds 10.

### Hierarchical ALS and soft orthogonality constraint

The standard ALS algorithm can be improved with respect to the convergence rate and performance by imposing additional constraints such as sparsity and smoothness. The update rules (5) and (6) are not computationally optimal because for large-scale problems the matrices **C** and **S** are enormous in size, which increases the time required for the inverse matrix calculation in each iterative step. This calculation can be avoided by using the hierarchical ALS (HALS) algorithm, which updates a smaller block of a column vector in **C** or **S** at each update [21,22]. Optimization of the block to minimize the approximation error can be analytically solved. Then the update rule is given by

(11) \begin{equation*} {\mathbf{C}}_{\bullet k}={\left[{\mathbf{X}}^{(k)}{\mathbf{S}}_{\bullet k}\right]}_{+}, \end{equation*}

(12) \begin{equation*} {\mathbf{S}}_{\bullet k}={\left[{\mathbf{X}}^{(k)\mathrm{T}}{\mathbf{C}}_{\bullet k}\right]}_{+}/\left\Vert{\left[{\mathbf{X}}^{(k)\mathrm{T}}{\mathbf{C}}_{\bullet k}\right]}_{+}\right\Vert, \end{equation*}

where ${\mathbf{C}}_{\bullet k}$ and ${\mathbf{S}}_{\bullet k}$**,** respectively, are the *k*-th column of **C** and **S**, ${\mathbf{X}}^{(k)}=\mathbf{X}-\mathbf{C}{\mathbf{S}}^{\mathbf{T}}+{\mathbf{C}}_{\bullet k}{\mathbf{S}}_{\bullet k}^{\mathrm{T}}$. The updates can be implemented faster than ALS under the assumption of *K* < < *N_xy_* and *K* < < *N*_ch_, which is a basic assumption of NMF.

The ALS and HALS methods generate sparse matrices for both **C** and **S**. This behavior does not cause any problem for STEM-EDXS HSIA where both spatial component distributions and their spectra are sparse and mostly do not overlap with one another. However, the amplitudes of spectra in STEM-EELS HSI data are non-zero for most energy loss channels, meaning that the spectra are not sparse, as mentioned in the previous section. Thus, for STEM-EELS HSI data, the existing NMFs often generate unnatural decomposition by biasing decomposed spectra to being sparse [15].

To overcome the above difficulties, we proposed an extended NMF model that imposes a spatial orthogonal constraint on **C** [23]. To alleviate the strict constraint of spatial orthogonality, we proposed the introduction of a weight parameter *w* for the constraint, known as a soft-orthogonal (SO) constraint. This optimization for ${\mathbf{C}}_{\bullet k}$ minimizes the following cost function:

(13) \begin{equation*} \underset{{\mathbf{C}}_{\bullet k}}{\min\ }{\left\Vert \mathbf{X}-\mathbf{C}{\mathbf{S}}^{\mathrm{T}}\right\Vert}_F^2+w\bullet{\xi}_k{\mathbf{C}}^{(k)\mathrm{T}}{\mathbf{C}}_{\bullet k}, \end{equation*}

where *w,*$0\le w\le 1$, is a parameter to adjust the orthogonal constraint, ${\mathbf{C}}^{(k)}={\sum}_{\boldsymbol{j}\boldsymbol{\ne}\boldsymbol{k}}{\mathbf{C}}_{\bullet k}$ and ${\xi}_k$ is the Lagrange multiplier for the exact orthogonal constraint of **C**. The optimization (13) can be analytically solved, after which the update can be implemented by matrix calculations [23]. When *w* = 1, the optimized **C** is an orthogonal matrix in which no chemical components overlap. When *w* = 0, the optimized components in **C** may extensively overlap. The optimal value of *w* depends on the situation, such as the spatial resolution of the data (step width of STEM-HSI) and localization of chemical states. Thus, the optimal value of *w* must be chosen according to the measurement level.

**Fig. 3:**
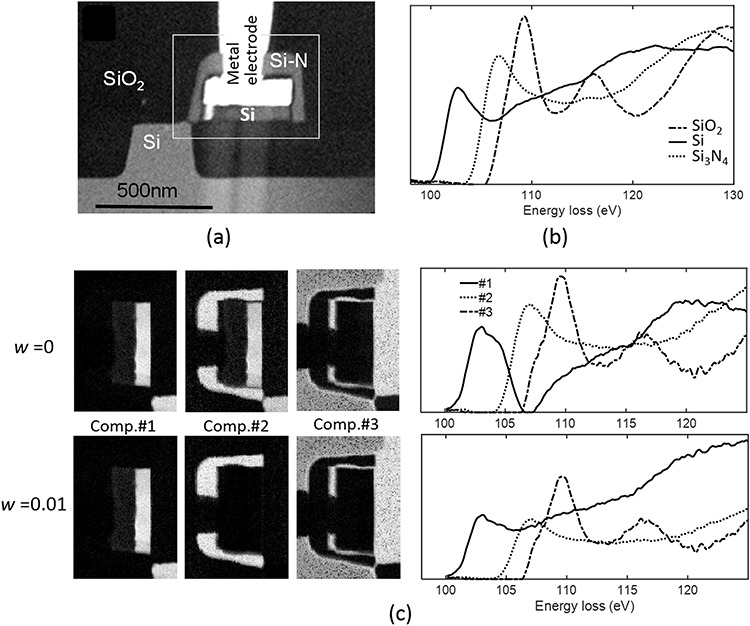
(a) ADF-STEM image of cross-sectional sample of a Si diode. (b) Si-L_2,3_ EEL spectra of the constituent parts in (a). (c) Isolated component spectra and their spatial maps obtained by applying NMF to STEM-EELS HSI data with *w* = 0 (upper row) and 0.01 (bottom row), respectively.

A real EELS data set was acquired from a cross-sectional TEM sample of a Si diode, prepared by a focused ion beam technique (Fig. 3a). We measured the HSI data for Si-L_2,3_. Figure 3a and b show the expected component construction and reference spectra, respectively. In this EELS-HSI, the three existing components are clearly separated spatially, i.e. an orthogonal matrix ***C*** would be expected as the reference distribution. On the other hand, their spectra are not orthogonal owing to the non-zero intensities with different peak positions.

Figure 3c shows the decomposition results obtained by NMF without the SO constraint (upper row: *w* = 0) and NMF-SO (bottom row: *w* = 0.01) with *K* = 3. The result of using NMF without SO exhibits an unnatural reduction in the intensity of the spectrum at 110 eV and the component maps generated by NMF are unnaturally overlapped, which is inconsistent with the results expected on the basis of Fig. 3c. In contrast, NMF-SO provides correct results that are almost consistent with Fig. 3c.

We have extensively evaluated our NMF-SO schemes using several EELS- and EDXS-HSI data sets and the results demonstrated the effectiveness of the above-mentioned approach [23,24].

### Optimization of the number of components

**Fig. 4:**
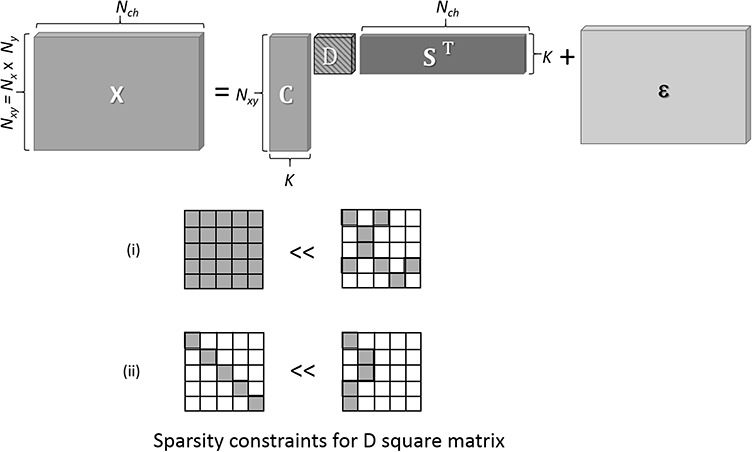
Conceptual illustration of ARD scheme. Square core matrix **D** is introduced for ARD optimization, where two additional constraints are imposed on **D**, as shown in (i) and (ii). Criterion (i): more appropriate for a more sparse solution. Criterion (ii): more appropriate for a smaller number of non-zero columns (equivalent to fewer components).

Existing NMFs require the number of spectral components in advance. Optimizing the number of components using only the given data is an important practical problem. Maximum likelihood estimations, which are equivalent to estimations based on minimizing errors, are ineffective in such cases because they monotonically increase as the number of components becomes larger, thereby causing the observed HSI data to experience overfitting. The problem of overfitting can be avoided by employing a Bayesian estimation [or maximum *a posteriori* (MAP) estimation], which introduces a prior distribution of scale parameters (relevance weights) to constrain the optimizing parameters [25]. The process whereby only the important components are chosen is known as automatic relevance determination (ARD). In general, ARD is performed by assuming a prior probability distribution that causes sparseness such as an exponential distribution. Our proposed NMF, which we named ARD-NMF, assumes that the probability density function of ${\mathbf{C}}_{\bullet k}$ is an exponential distribution with the scale parameter ${\lambda}_k$. The probability density function of ${\lambda}_k$is assumed by using an inverse Gamma distribution function with a few hyper-parameters [25]. This assumption is essentially the same as that of Dobigeon et al. [26] to generate sparse **C** and to choose only a small number of vectors. The principle underlying the ARD implementation is schematically illustrated in Fig. 4 and the full description is given elsewhere [23].

**Fig. 5:**
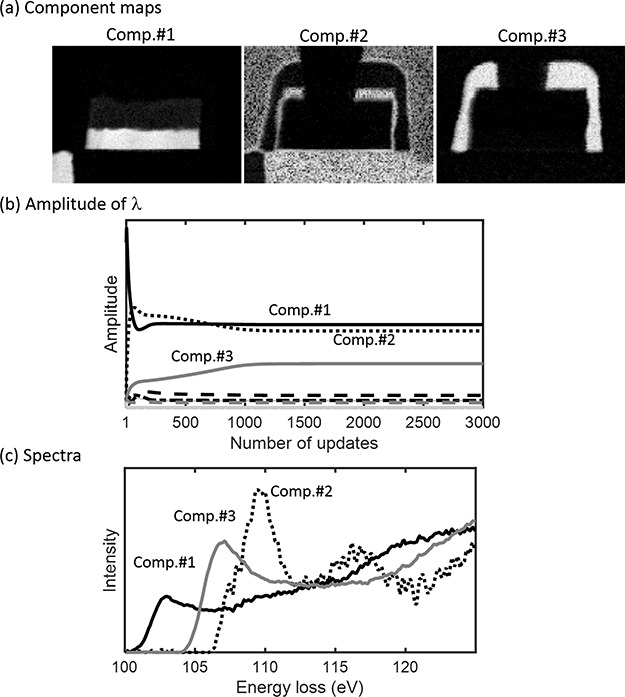
Result of ARF-SO-NMF (*w* = 0.01) for Si-*L*_2,3_ STEM-EELS-HSI data from silicon diode sample.

We applied our proposed ARD-SO-NMF to this EELS-HSI data set starting with *K* = 10 and with the SO penalty (*w* = 0.01). Figure 5 shows the (a) generated component maps, (b) their spectra and (c) the amplitude of components ${\lambda}_k$, *k* = 1, …, *K*. Figure 5c shows that, even if the initial number of components was large, only the essential components survived after the optimization. This result demonstrated that our ARD was able to choose only the essential components, which are the same as the reference components. The component distributions generated by ARD-SO-NMF and shown in Fig. 5a do not overlap; thus, the correct solution is provided.

The ARD-SO-NMF scheme has not yet been fully tested for detecting very weak signals of which the intensity barely exceeds the noise level. NMF users are thus advised to crosscheck the conceivable value of *K* using a combination of other well-known empirical methods, such as the standard Scree plot [4] criterion and Malinowski’s factor indication function [27] based on SVD. The effects of including noise on the PCA analysis were discussed before [28,29].

### Signal subspace sampling preprocess

**Fig. 6:**
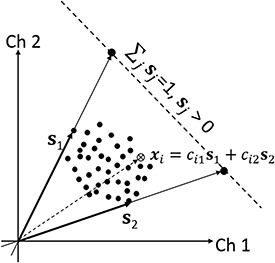
2D schematic illustration of key concept of VCA for 2-channel detector: each experimental point *x* should be expressed by a linear combination of latent basis vectors, **s**_1_ and **s**_2_ (pure components included in the data), which are supposed to be the outermost points if appropriately projected. The final basis vectors excluding the scale ambiguity are the points of intersection between **s***_j_* and the simplex condition (${\sum}_j{\mathbf{s}}_j$=1, ${\mathbf{s}}_j$ > 0) line. The VCA algorithm searches such basis vectors to subtend the triangle including all the data points, subsequently projected onto the subspace [21].

An alternative and efficient algorithm free from the non-uniqueness problem is vertex component analysis (VCA), where the **C** matrix is assumed to include ‘pure pixels’, which consist of a single spectral component [30,31]. The VCA algorithm iteratively projects data onto a direction orthogonal to the subspace spanned by the spectral components already determined. The new component signature corresponds to the extreme of the projection, and the algorithm iterates until the number of endmembers (spectral components) is exhausted. The VCA principle is schematically illustrated in Fig. 6.

All spectroscopic techniques in electron microscopy are problematic in that the signals generated by the sample appear strongly mixed: core-loss spectra in EELS overlap, characteristic X-ray emission peaks at low energies in EDXS may not be fully separated, and, more commonly, the electron beam passes through different phases in the sample such that the signal measured at a given pixel in the measured spectrum image is acquired as the superposition of overlapped signals. As mentioned above, the solution provided by VCA is unique if the pure-pixel assumption holds and the non-negative source spectra are orthogonal or non-overlapping in at least one channel. This assumption, however, is often not fulfilled experimentally; consequently, suitable data pre-processing has to be applied to extract the desired information from the experimental data.

Spiegelberg et al. [32] developed a novel pre-processing scheme for VCA named signal subspace sampling (SSS). Although the pure-pixel assumption is often violated in experimental data, the second condition of partial orthogonality can be assumed for certain signal classes. A strategy toward unique NMF in the absence of pure pixels in the raw data is to sample the signal subspace while enforcing non-negativity. The key concept behind this subspace sampling step is to artificially generate pure pixels: every point in the signal subspace can be expressed as a linear combination of the true source components (cf.: *i*-th vector ***s****_i_* in Fig. 6), but the source components are known to be non-negative and at least partially non-overlapping. In practice, the subspace can be conveniently accessed by using PCA and sampling can be achieved by randomly generating artificial scores and saving those that meet the non-negativity criterion while rejecting the others. In addition to non-negativity, we assume that the source signals corresponding to the different phases of the sample differ in at least one peak (but are otherwise strongly overlapped), which is a natural constraint for X-ray emission (EDXS) signals. After plotting sufficiently many data points, one can safely assume that pure source spectra fulfilling the above conditions are present in the sampled data set. The conventional VCA method can then be used to efficiently extract the pure source spectra.

In a successful example [32], the SSS + VCA strategy was applied to the plan-view of an Al/Fe/Si_3_N_4_ multilayer and enabled each single layer to be isolated from the overlapped STEM-EDXS data set. The same scheme is also applicable to unmixing strong spectral overlap only in STEM-EELS HSI data by SSS pre-processing on the abundance **C** matrix instead of **S** [32], because the cost function (9) is symmetric with respect to **C** and **S**^T^.

A set of geometric data decomposition methods was found to be effective for application to noisy data sets and this has been intensively discussed [33].

### Extension from matrix to tensor

**Fig. 7:**
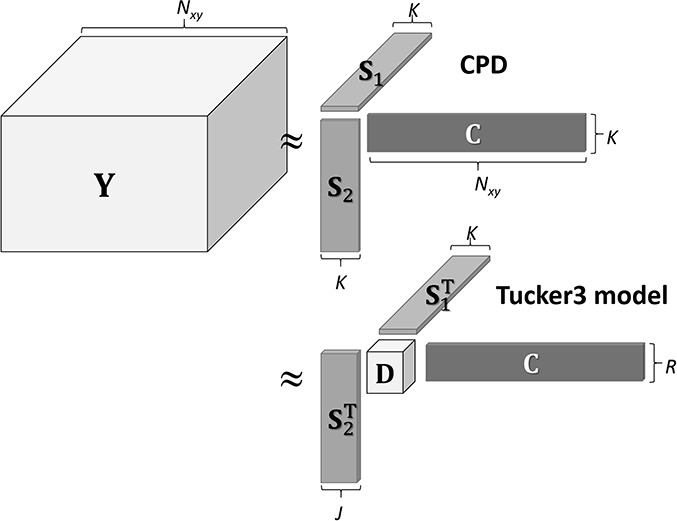
Schematic illustration of two representative tensor decomposition schemes: canonical polyadic decomposition (CPD) and the Tucker 3 model for a third-order tensor, which are extensions of Figs. 2 and 5, respectively. If *K* = *J* = *R*, **D** is a diagonal tensor, and the Tucker 3 model is equivalent to CPD.

Modern STEM measurement acquisition platforms allow for several different signals, such as annular dark-field images, EELS, EDXS and cathodoluminescence (CL) signals, to be concurrently acquired at every measured pixel when the electron beam is swept across the ROI without reducing the scanning speed, as shown in Fig. 1. From a data processing perspective, the increased information obtained should clearly yield a more complete picture of the sample, which would usually be achieved by separately analyzing the data acquired by different detectors. Because all detectors measure signals from a single pixel, the spatial structures of the corresponding source components are likely to be highly correlated. We investigated the way in which inter-set correlations can be exploited by jointly processing them using a recent BSS technique, namely tensor decomposition [34] and data fusion approaches [termed ‘structured data fusion’ (SDF)] [35] for electron microscopy. This represents an extension of the matrix factorization (Figs. 2 and 4) explained in the preceding sections to tensor factorization (as schematically shown in Fig. 7 for a third-order tensor). The proposed framework, which enables different signals from multiple detectors to be processed, assigns a different kind of signal to the entries of the tensor with different indexes. In addition, the SDF framework allows you to impose additional constraint conditions such as assigning fixed values to some components, correlative coupling between different types of spectroscopic data [36] and smoothing the spatial distributions of endmembers without being conscious of the mathematically complicated tensor construction [37]. The coupling between factorizations and the structure imposed on the factors can all be chosen freely without any changes to the solver, the platform of which is provided as a MATLAB toolbox [38]. Readers can find more useful and intriguing applications of tensor decomposition to STEM-EDXS/EELS HSIA in the literature [39–42].

### SDF for concurrently obtained EELS and EDXS data

The example system we selected to highlight the use of data fusion for extracting source components was a coupled high-angular-resolution electron-channeling X-ray/electron spectroscope (HARECXS/HARECES) that was used to analyze the Li_0.2_Ni_0.7_Mn_1.6_O_4_ system [39]. A series of EEL and EDXS spectra were recorded by tilting the sample at different angles. This changed the weights of the signals of Mn occupying different crystallographic sites such that the two species could be analyzed separately. Previously, a tedious procedure was employed to use unmixing to separate the contributions of the two different Mn species [43]. This procedure determined the extent to which Mn occupied the two different sites from the HAREXCS data and then solved the matrix Eq. (2) for the HARECES data by using the occupancies and theoretical prediction of the transition probabilities for EELS. Here, we address this source separation problem using SDF.

When the use of SDF is attempted to achieve unmixing, it is necessary to include additional constraints to improve the results by minimizing the following matrix function:

(14) \begin{equation*} {D}_F\left(\left.\mathbf{X}\right\Vert \mathbf{C}{\mathbf{S}}^{\mathrm{T}}\right)={\left\Vert{\mathbf{X}}_1-\left(\mathbf{C}+\mathbf{N}\right){\mathbf{S}}_1^{\mathrm{T}}\right\Vert}^2+\lambda{\left\Vert{\mathbf{X}}_2-\mathbf{C}{\mathbf{S}}_2^{\mathrm{T}}\right\Vert}^2+\gamma{\left\Vert \mathbf{N}\right\Vert}_{\mathrm{F},} \end{equation*}

where **X**_1_ and **X**_2_ are experimental EEL and EDXS spectra, **C** + **N** and **C** are the weights of the signals corresponding to the EELS and EDXS signal components, respectively, *λ* and *γ* are appropriate weights set according to the significance of each term, and **N** expresses weak coupling between the weights of the EELS and EDXS signals under the same illumination condition. From a physical point of view, this process directly enables the common site occupancies and the weakly coupled electronic transition probabilities for EELS and EDXS, respectively, to be predicted [39].

**Fig. 8:**
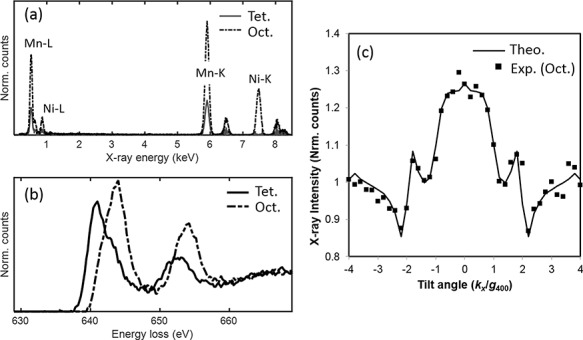
Source components recovered using SDF on the coupled HARECXS/HARECES data set. (a) EDXS source spectra and (b) EEL source spectra. (c) Experimental (estimated by SDF) and theoretical normalized Mn (octahedral site) K-edge intensity of the X-ray pattern as a function of the tilt angle. The theoretical curve is simulated by assuming tetragonal site occupancy of 0.35, consistent with the conventional analysis result [43].

The resulting source components are displayed in Fig. 8a. An excellent resemblance to the reference spectra of Mn^2+^ and Mn^3+^ [43] is achieved. Thus, using SDF, we were able to separate the contributions of Mn into different oxidation states without requiring reference spectra. Further analysis of the weights of the tilt series to determine the occupancy at divalent and trivalent sites showed excellent agreement with the theoretical results [39], as shown in Fig. 8b.

### Pre-edge background in ELNES: to be or not to be

Spectroscopic data usually include background intensities, e.g. continuous intensities at the low-energy side mainly due to Bremsstrahlung in EDXS and a smoothly decaying pre-edge background obeying the power law in EELS. In most cases, the near-edge fine structure (ELNES) of the core-loss spectrum is of interest and the pre-edge background would be subtracted in advance before NMF is applied. Otherwise, the algorithm may isolate many components related to subtle features included in the background profile particularly for data with low SNR, because the pre-edge background comprises the majority of the spectral intensities and its profile depends on the phase or chemistry at the sampling points. Pre-processing the data in this manner could always be criticized: from a statistical point of view, pre-edge background subtraction creates zero entries in the background region, which imposes an unnatural bias to the data because the non-negative constraint no longer makes sense under the condition for which Gaussian noise is assumed. Whether the background should be subtracted in advance before the main NMF code is applied therefore remains controversial. In principle, it is advisable to process the data without subtracting the background because the background intensities convey physical information of the sampling points.

**Fig. 9:**
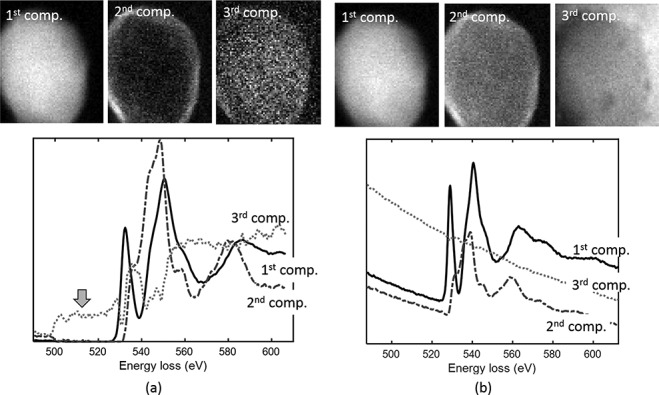
Isolated O-K ELNES and spatial map of each component by 3-component NMF analysis of a particle of LIB active cathode material, Li (Mn, Co, Ni)_2_O_4_. (a) Conventional NMF applied to the data with the pre-edge background subtracted. (b) Pre-edge background taken into account in the framework of SDF, based on Eq. (15).

On the other hand, particularly in EELS, the pre-edge background intensity is comparable with or sometimes even larger than the net core-loss intensity, which results in the NMF solution being partially biased or more sensitive to background variations rather than to the genuine spectral fine structure. An example is shown in Fig. 9, where the two components of O-K ELNES were extracted from the STEM-EELS-HSI data set of a representative particle of the cathode material [Li (Mn,Co,Ni)_2_O_3_] of a lithium-ion battery (LiB). In this case, the pre-edge background was subtracted before NMF was applied. The corresponding abundance maps, which are shown in Fig. 9a, exhibit the spatial distributions of the pristine, degraded phases and an additional oxygen component that presumably originates from the resin in which the particle is embedded. Note that the second component exhibited a positively biased intensity in the pre-edge background region owing to the non-negativity constraint. It is not always obvious where the spectral biases are reflected in abundance maps.

For this purpose, the descriptor to be minimized was that in Eq. (15), which is mathematically equivalent to Eq. (14), but the roles of **S** and **C** are interchanged. Thus, the first Euclidian norm includes the experimental data set without subtracting the background as **X**_1_, and **N** models the background by imposing the additional constraint of a smooth and monotonically decaying (e.g. power law) function [40]. The second norm also includes the experimental data but with the background subtracted:

(15) \begin{equation*} {D}_F\left(\left.\mathbf{X}\right\Vert \mathbf{C}{\mathbf{S}}^{\mathrm{T}}\right)={\left\Vert{\mathbf{X}}_1-\left(\mathbf{S}+\mathbf{N}\right){\mathbf{C}}^{\mathrm{T}}\right\Vert}^2+{\left\Vert{\mathbf{X}}_2-\mathbf{S}{\mathbf{C}}^{\mathrm{T}}\right\Vert}^2+\gamma{\left\Vert \mathbf{N}\right\Vert}_{\mathrm{F}}. \end{equation*}

This time, the O-K ELNES unfolded, as shown in Fig. 9b. It should be noted that the modified model provided an additional third significant component other than the pristine and degraded phases. This component corresponds to the epoxy resin in which the sample is embedded and is derived from the post-edge background of carbon and nitrogen extending to the higher energy side and including a slight amount of oxygen. The unnatural biased spectral intensity no longer appeared in the pre-edge region.

Another modern data compression method for pre-edge background subtraction was introduced by Spiegelberg et al. [41].

## Special remarks on noise treatment

NMF/NTF finds a set of non-negative matrices **C** and **S** by minimizing ${\Big\Vert \mathbf{X}-\mathbf{C}{\mathbf{S}}^{\mathrm{T}}\Big\Vert}^2$ such that the residual matrix **E** in Eq. (2) contains statistical noise alone. In general, BSS techniques assume Gaussian and Poisson noise. It should thus be noted that **C** and **S** would be contaminated or irrelevantly biased if the data matrix **X** contained systematic noise that was not mathematically assumed in the formulation because the process performs computations on the data as the sum of signals and statistical noise, as shown in a typical example in Fig. 9a. EELS, mostly when using a charge-coupled device (CCD) detector, is supposed to detect read-out noise, photon conversion noise, gain noise, spike noise and residual signals due to afterglow other than statistical noise, all of which are overlaid upon each other. In this respect, NMF users should first screen these systematic noise sources as a pre-processing step before the main algorithm is applied, particularly for low SNR data.

NMF effectively screens a certain type of noise pattern besides the statistical noise: this is known as the video-background subtraction problem [44]. The static background of the frames has a low-rank property because it changes slightly, and the dynamic foreground can be regarded as sparse components in the frames. In a similar sense, the regular noise pattern on the CCD channels, such as the sparse components, can be effectively screened out by shifting (wobbling) the spectrum on the detector during SI data acquisition [45] (this can be achieved by using the ‘SpectrumWobbler’ script [46] for background processing in the control software Gatan Microscopy Suite), followed by applying NMF to the aligned SI data.

Noteworthy is also that the relation between the experimental SNR and detectability of trace components in PCA is discussed in [28,29].

## Closing remarks

In this review article, we introduced selected HSIA strategies for unmixing multiband spectral data to separate the data into their latent pure components and spatial maps. These strategies are designed to be particularly effective for STEM-EELS/EDXS HSI data, although the schemes should be widely applicable to similar spectroscopic data sets such as those generated by 3D atom probe microscopy, Raman/FTIR microscopy and secondary ion mass spectroscopy, by flexibly imposing appropriate constraints depending on the characteristics of the data. We would emphasize that the provided solution could never be relevant insofar as a mathematical model such as Eq. (4) may not correctly reflect the data characteristics. It is thus essential to modify or customize existing methods or available algorithms, rather than simply applying one of them as a black box to process your own data. In this respect, users require deep insight to obtain an approximate impression of the ‘correct’ solution by developing their own intuitive judgment criteria and by accumulating solid experience of data analysis. In this sense, the HSIA schemes should be recognized as convenient instead of foolproof ‘tools’. Particularly, quantification requires users to exercise the highest caution and they need to experiment with multiple methods to crosscheck the consistency and physical plausibility of the solution.

Recently emerged image/data reconstruction techniques are able to perform ‘compressive sensing’, in which the proposed random sampling schemes successfully provide highly accurate image recovery results from only partial information [47]. The techniques, which are particularly useful and effective for dynamical time-dependent measurements, reduce the electron dose in electron-sensitive materials, which is beyond the scope of the present review.

Information in science should be based on measurements. A measured value is the projection of reality by using a detector, which is a convolution of the instrumental function and the true value plus noise. Ongoing increases in the processing speed and memory capacity of computers enables increasingly larger amounts of data to be processed, thereby heralding in the current era of ‘big data’. However, it would be too convenient to think that AI could be relied upon for everything, as far as we attempt to unravel the secrets of nature instead of fighting in the world of games where all possible candidates or strategies are comprehensively sought within the specified operational space under clearly defined rules.

Ultimately, machine learning should be considered as being complementary to human abilities; it should be kept in mind that users should continue their steady efforts to improve the quality of data and examine intensively whether the solution provided by the computer corresponds to their physical or empirical intuition without accepting it uncritically if they really intend obtaining trustable results by utilizing these tools. It would be the same as putting the cart before the horse if the development of these useful computer tools would lead to a gradual decline in human abilities and the knowledge incessantly accumulated by pioneers’ wisdom and efforts. We would like to additionally remark here that researchers could expect to obtain unexpected spinoffs by using this approach to data analysis that would deepen their fundamental understanding of scientific measurements, namely to understand the characteristics of noise and to strive to improve the quality of data.

From an appropriate perspective, the emergence of ‘data-driven science’ beyond the conventional analysis methods could be envisioned to have transformed from being a historical necessity accelerated by the current trends of various database services and instrumental automation, the power of which can be superb, if appropriately utilized. We hope that many researchers in this field could share in its benefits and that the advice in this article is of assistance.

## Funding

Grants-in-Aid for Scientific Research on Innovative Areas ‘Nano Informatics’ (grants no. 25106004, 26106510 and 16H00736); ‘Interface Ionics’ (grant no. 19H05815); KIBAN-KENKYU A (grant no. 26249096); B (grant no. 16H02866) from the Japan Society for the Promotion of Science; and by Precursory Research for Embryonic Science and Technology (grant no. JPMJPR16N6) from Japan Science and Technology Agency.
